# Coronakinderstudien „Co-Ki“: erste Ergebnisse eines deutschlandweiten Registers zur Mund-Nasen-Bedeckung (Maske) bei Kindern

**DOI:** 10.1007/s00112-021-01133-9

**Published:** 2021-02-22

**Authors:** Silke Schwarz, Ekkehart Jenetzky, Hanno Krafft, Tobias Maurer, David Martin

**Affiliations:** 1 Fakultät für Gesundheit/Department für Humanmedizin, Universität Witten/Herdecke, Witten/Herdecke, Deutschland; 2 Klinik und Poliklinik für Kinder- und Jugendpsychiatrie und -psychotherapie, Universitätsmedizin Mainz, Mainz, Deutschland; 3 Universitätsklinik für Kinder- und Jugendmedizin, Universität Tübingen, Tübingen, Deutschland

**Keywords:** Mund-Nasen-Schutz, Alltagsmasken, Maskenpflicht, Pädiatrie, COVID-19, Mouth and nose protection, Community masks, Mask obligation, Pediatrics, COVID-19

## Abstract

**Hintergrund:**

Bei Kindern- und Jugendlichen häufen sich Narrative über Beschwerden durch das Tragen eines Mund-Nasen-Schutzes (Maske). Weltweit existiert bisher kein Register für mögliche Nebenwirkungen von Masken.

**Methode:**

Im Rahmen des www.Co-Ki.de Multi-Studienkomplexes wurde ein Online-Register aufgebaut, im dem Eltern, Ärzt*innen, Pädagog*innen und andere ihre Beobachtungen zu den Auswirkungen des Tragens einer Maske bei Kindern und Jugendlichen eintragen können. Am 20.10.2020 wurden 363 Ärzt*innen eingeladen, Eintragungen zu tätigen und auf das Register hinzuweisen.

**Ergebnisse:**

Bis zum 26.10.2020 hatten 20.353 Personen an der Umfrage teilgenommen. Allein die Gruppe der Eltern gab Daten zu 25.930 Kindern ein. Die angegebene durchschnittliche Tragedauer der Maske lag bei 270 min am Tag. Die Eingebenden berichten zu 68 %, dass Kinder über Beeinträchtigungen durch das Maskentragen klagen. Zu den Nebenwirkungen zählten Gereiztheit (60 %), Kopfschmerzen (53 %), Konzentrationsschwierigkeiten (50 %), weniger Fröhlichkeit (49 %), Schul‑/Kindergartenunlust (44 %), Unwohlsein (42 %), Beeinträchtigungen beim Lernen (38 %) und Benommenheit/Müdigkeit (37 %).

**Diskussion:**

Dieses weltweit erste Register zur Erfassung von Auswirkungen des Tragens eines Mund-Nasen-Schutzes bei Kindern und Jugendlichen widmet sich einer neuen Forschungsfrage. Eine Verzerrung im Hinblick auf die präferenzielle Dokumentation besonders schwer betroffener Kinder oder den Schutzmaßnahmen grundsätzlich kritisch gegenüberstehenden Personen lässt sich nicht ausschließen. Die Nutzungshäufigkeit und das Symptomspektrum weisen auf die Wichtigkeit des Themas hin und rufen nach repräsentativen Surveys, randomisierten kontrollierten Studien mit verschiedenen Maskensorten und nach einer Nutzen-Risiko-Abwägung der Maskenpflicht bei der vulnerablen Gruppe der Kinder.

## Abstract (en)

**Background:**

Narratives about complaints in children and adolescents caused by wearing a mask are accumulating. There is, to date, no registry for side effects of masks.

**Methods:**

In the context of the www.co-ki.de multi-study complex, an online registry has been set up where parents, doctors, pedagogues and others can enter their observations. On 20 October 2020, 363 doctors were asked to make entries and to make parents and teachers aware of the registry.

**Results:**

By 26 October 2020, a total of 20,353 people had taken part in the survey. The group of parents alone entered data on a total of 25,930 children. The average reported wearing time of masks was 270 min per day. Of the respondents 68% reported that children complained about impairments caused by wearing the mask. Side effects included irritability (60%), headache (53%), difficulty concentrating (50%), less happiness (49%), reluctance to go to school/kindergarten (44%), malaise (42%) impaired learning (38%) and drowsiness/fatigue (37%).

**Discussion:**

This world’s first registry for recording the effects of wearing masks in children is dedicated to a new research question. A bias with respect to the preferential documentation of particularly severely affected children or persons who are fundamentally critical of protective measures cannot be ruled out.

The frequency of use and the spectrum of symptoms registered indicate the importance of the topic and call for representative surveys, randomized controlled trials with various masks and a renewed risk-benefit assessment of mask obligation in the vulnerable group of children.

## Hintergrund und Fragestellung

Die 2020 in Deutschland zur Eindämmung der COVID-19-Pandemie empfohlene Kombination von Vorsorgemaßnahmen, kurz AHA-L(Abstand/Hygiene/Alltagsmaske/Lüften)-Regel, trägt einen wesentlichen Beitrag zur Eindämmung des Infektionsgeschehens bei. Eltern, Pädagog*innen und Ärzt*innen berichten bei Kindern zunehmend von Problemen und gesundheitlichen Beschwerden, die im Zusammenhang mit dem Tragen eines Mund-Nasen-Schutzes – im Folgenden „Maske“ genannt – gesehen werden. Die Frage nach einem Attest zur Befreiung von der Maskenpflicht ist ein neues Phänomen in der pädiatrischen Praxis. Zum Einsatz bei Kindern und Jugendlichen gibt es zu Masken, welche im beruflichen Einsatzgebieten als Medizinprodukt zum Arbeitsschutz zertifiziert sind, keine herstellerunabhängigen Studien. Zudem existieren zu den, vermutlich von der Mehrheit der Kinder getragenen, „Alltagsmasken“ aufgrund der verwendeten unbekannten Materialien, keinerlei Erkenntnisse zu Nebenwirkungen bei längerer Nutzung. In Anbetracht der anhaltenden Maßnahmen zur Eindämmung der COVID-19-Pandemie und insbesondere der weitreichenden Verpflichtung des Tragens von Masken bei Kindern und Jugendlichen im Schulunterricht über längere Zeit, besteht daher dringender Forschungsbedarf.

## Studiendesign und Untersuchungsmethoden

**Figure 1:**
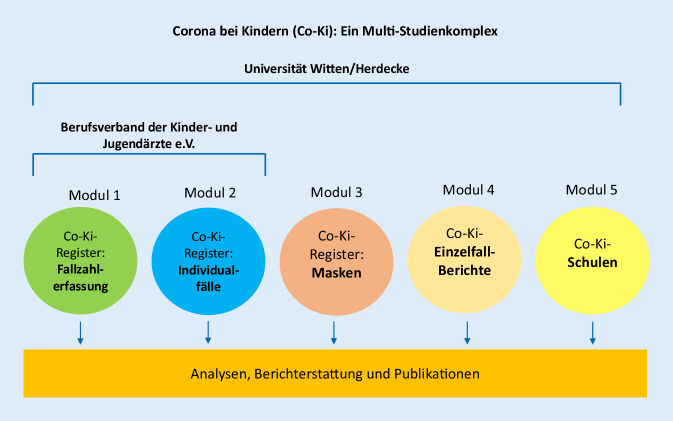

In Anlehnung an das Register der Nebenwirkungen von Arzneimitteln am Paul-Ehrlich-Institut (www.nebenwirkungen.pei.de) wurde ein Online-Register aufgebaut, im dem Eltern, Ärzt*innen, Pädagog*innen und andere Personen ihre Beobachtungen zu den Auswirkungen des Tragens einer Maske bei Kindern und Jugendlichen eintragen können. Am 20.10.2020 wurden die 363 Ärzt*innen des Co-Ki-Studienverteilers über die Möglichkeit informiert, dort selbst Eintragungen zu tätigen und darauf hinzuweisen. Das Register sowie der Fragebogen sind online auf der Webseite www.co-ki-masken.de zu finden, als Teil des Co-Ki-Studienkomplexes (Abb. 1).

Die ins Register eingetragenen Daten umfassen Informationen bezüglich der Rolle des Eingebenden, demografische Daten, Vorerkrankungen, Situation und Dauer des Maskentragens, Art der Maske, Vorhandensein von Klagen des Kindes zu einer Beeinträchtigung über die Maske, Symptomatik, Verhaltensauffälligkeiten, die persönliche Haltung zu den Coronaschutzmaßnahmen der Regierung der Eintragenden und die Möglichkeit, Namen und Mailadresse zu hinterlassen. Ein Ethikvotum der Universität Witten/Herdecke liegt vor.

Ziel dieser ersten Erhebung ist es, subjektive Beschwerden niederschwellig absolut zu quantifizieren und inhaltlich zu klassifizieren. Dies geschieht durch den Bericht von absoluten und relativen Häufigkeiten. Die Verteilung von Geschlecht, Bundesland oder Alter mit bekannten Erwartungswerten gibt erste Hinweise auf die quotierte Repräsentativität des Antwortverhaltens. Mithilfe von explorativen *p*-Werten durch den Chi^2^-Test werden die Häufigkeiten in den 3 gewählten Altersgruppen verglichen. Bei der Tragezeit in Minuten kam der Kruskal-Wallis H-Test zur Anwendung. Eingeschlossen wurden die Antworten aller teilnehmenden Personen. Ausgeschlossen wurden unvollständige Antworten sowie offensichtliche Falscheingaben. In dieser ersten Analyse werden nur die Antworten aus der größten Gruppe der „Eltern“ betrachtet.

## Ergebnisse

**Figure 2:**
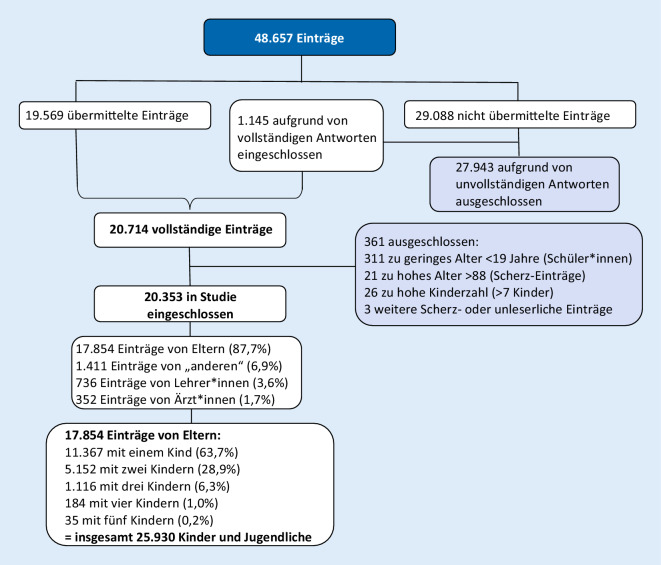

Am Abend des 26.10.2020 hatten bereits 20.353 Personen an der Umfrage teilgenommen. Von den Umfrageteilnehmern waren 17.854 (87,7 %) Eltern, 736 (3,6 %) Lehrer*innen, 352 (1,7 %) Ärzt*innen und 1411 (6,9 %) „andere“. Zur Auswertung der insgesamt 48.657 Einträge kamen die bereinigten, vollständigen 20.353 Datensätze (41,8 %) von teilnehmenden Eltern, Ärzt*innen und Lehrer*innen. (Den Analysesatz der Einträge der ersten Woche zeigt Abb. 2.) Von den 17.854 eingebenden Eltern (87,7 %) mit insgesamt 25.930 Kindern und Jugendlichen berichtet dieser Artikel. Die Daten der eingebenden Ärzt*innen, Lehrer*innen und anderer Rollen werden separat veröffentlicht.

**Figure 3:**
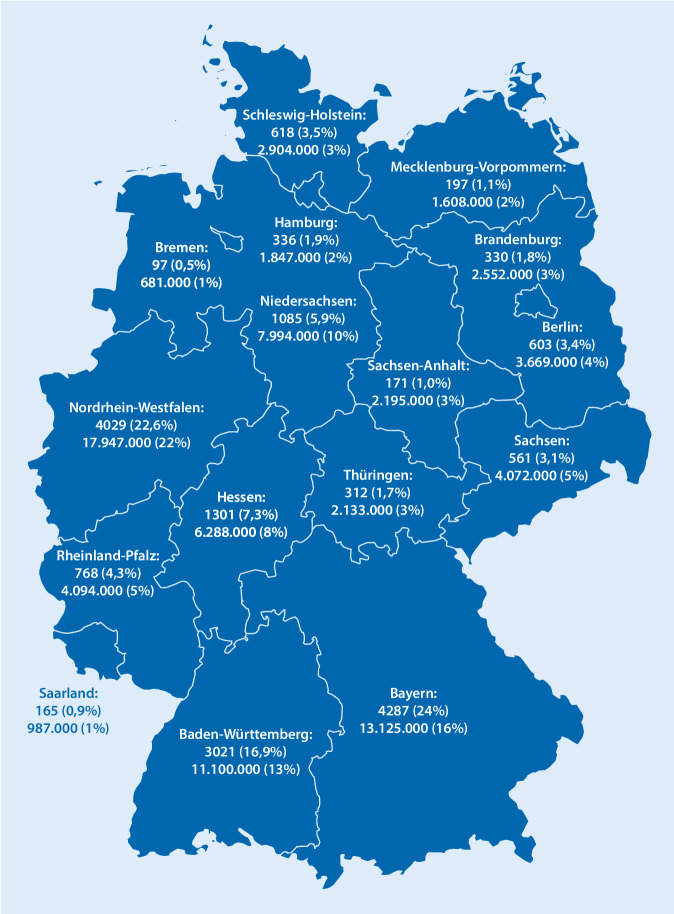

Von den 17.854 eintragenden Eltern gaben 6877 (38,5 %) an, einen (Fach‑)Hochschulabschluss (Bachelor, Master, Magister, Diplom, Staatsexamen, Promotion) zu haben, 671 (3,8 %) haben einen Meister, 3704 (20,7 %) eine abgeschlossene Lehre und 3040 (17,0 %) Abitur (allgemeine Hochschulreife) bzw. fachgebundene Hochschulreife oder Fachhochschulreife. 2509 (14,1 %) der Teilnehmenden gaben als höchsten Bildungsabschluss einen Realschulabschluss (mittlere Reife, Fachoberschulreife o. Ä.) an, 327 Teilnehmer (1,8 %) haben einen Hauptschulabschluss, 31 Teilnehmer (0,2 %) gaben an, keinen Schul- oder Ausbildungsabschluss zu haben. Der Rest machte keine Eingabe zur Bildung. Die Beteiligung der Eingebenden pro Bundesland entspricht in etwa der Verteilung der Bevölkerung (Abb. 3).

Die demografische Situation der Kinder und Vorerkrankungen bei den Kindern aus Elternsicht sind in Tab. 1 dargestellt. Von den 25.930 durch Eltern eingegebenen Kindern sind 12.248 (47,2 %) Mädchen und 12.589 (48,5 %) Jungen, bei 62 (0,2 %) wurde als Geschlecht „divers“ angegeben, bei 1031 (4 %) erfolgte keine Angabe. 55,6 % der Kinder waren im Alter zwischen 7 und 12 Jahren. Bei 79,4 % der Kinder wurde angegeben, dass sie keinerlei Vorerkrankungen hatten, 5,9 % hatten Asthma und 1,8 % eine andere Lungenerkrankung, des Weiteren gab es die Möglichkeit von Freitexteingaben zu weiteren Vorerkrankungen, die von 8,6 % genutzt wurde.

**Table 1:** 

		Gesamt, *n* (%)	Altersgruppe 0 bis 6 Jahre, *n* (%)	Altersgruppe 7 bis 12 Jahre, *n* (%)	Altersgruppe 13 bis 17 Jahre, *n* (%)	Chi^2^-Test auf Unterschied
–	Altersgruppe, *n* (%)	25.926^a^ (100)	4002 (15,4)	14.407 (55,6)	7517 (29,0)	–
Geschlecht	Männlich	12.589 (48,5)	1880 (47,0)	7027 (48,8)	3682 (49,0)	*p* = 0,0002
	Weiblich	12.248 (47,2)	1899 (47,5)	6790 (47,1)	3559 (47,3)	
	Divers	62 (0,2)	13 (0,3)	32 (0,2)	17 (0,2)	
	Ohne Angabe	1031^a^ (4,0)	210 (5,2)	558 (3,9)	259 (3,4)	
Vorerkrankungen	Keine Vorerkrankungen	20.586 (79,4)	3400 (85,0)	11.606 (80,6)	5580 (74,2)	*p* < 0,0001
	Asthma	1541 (5,9)	123 (3,1)	801 (5,6)	617 (8,2)	
	Andere Lungenerkrankungen	458 (1,8)	50 (1,2)	251 (1,7)	157 (2,1)	
	Andere Vorerkrankungen (Freitexteingaben)	2232 (8,6)	233 (5,8)	1178 (8,2)	821 (10,9)	
	Keine Angabe	1110 (4,3)	196 (4,9)	571 (4,0)	343 (4,5)	

^a^Für 4 Kinder fehlt die Altersangabe

Die Tragesituation von Masken bei den Kindern und ob irgendeine Form von Beeinträchtigung aus Elternsicht überhaupt vorhanden war, zeigt Tab. 2. So gaben beispielsweise 26 % der Eltern an, bei den Kindern keinerlei Beeinträchtigung wahrzunehmen. Auf die Frage nach den Situationen, in denen die Kinder eine Maske tragen, antworteten 81,1 % der Umfrageteilnehmer, dass das Kind die Maske in der Schule außerhalb der Klasse trägt, also in Pausen und auf den Fluren, 48,6 % gaben zudem an, dass das Kind die Maske auch in der Klasse am Sitzplatz während des Unterrichtes trägt. 68,5 % der erfassten Kinder tragen die Maske in Geschäften und 39 % auf dem Schulweg; 4,6 % der Kinder tragen nie eine Maske. Einige ältere Kinder tragen eine Maske bei Abholung der Geschwister im Kindergarten. Eine Maskenbefreiung (Attest) hatten 6,7 % der in der Umfrage erfassten Kinder. Die durchschnittliche Tragedauer der Maske variiert altersbezogen stark; sie lag im Mittel bei 4,5 Std. am Tag, gerade bei den älteren Kindern (13 bis 17 Jahre) mit durchschnittlich 6 Std. wesentlich höher (Tab. 2). Bei 16.913 Kinder (65,2 %) wurde angegeben, dass Stoffmasken getragen werden, gefolgt von OP-Masken.

**Table 2:** 

	Tragesituation		Gesamt, *n* (%)	Altersgruppe 0 bis 6 Jahre, *n* (%)	Altersgruppe 7 bis 12 Jahre, *n* (%)	Altersgruppe 13 bis 17 Jahre, *n* (%)	Chi^2^-Test auf Unterschied
–	Tragezeit in Minuten (IQR)		270 (120;390)	90 (30;240)	240 (120;370)	360 (240; 450)	*p* < 0,0001
Maskentyp	Stoffmaske		16.913 (65,2)	2501 (62,5)	10.311 (71,6)	4101 (54,6)	*p* < 0,0001
	OP-Maske		5.542 (21,4)	271 (6,8)	2619 (18,2)	2652 (35,3)	
	FFP-Maske		536 (2,1)	51 (1,3)	273 (1,9)	212 (2,8)	
	Keine Angabe		2935 (11,3)	1179 (29,5)	1204 (8,4)	552 (7,3)	
Beeinträchtigungen	Beeinträchtigung durch Maske laut Kind	Ja	17.550 (67,7)	1607 (40,2)	10.244 (71,1)	5699 (75,8)	*p* < 0,0001
		Nein	6801 (26,2)	1431 (35,8)	3744 (26,0)	1626 (21,6)	
		Keine Angabe	1575 (6,1)	964 (24,1)	419 (2,9)	192 (2,6)	
	Wurden Beeinträchtigungen beim Kind durch eine Maske beobachtet?	Ja	17.125 (66,1)	1640 (41,0)	9980 (69,3)	5505 (73,2)	*p* < 0,0001
		Nein	6841 (26,4)	1367 (34,2)	3810 (26,4)	1664 (22,1)	
		Keine Angabe	1960 (7,6)	995 (24,9)	617 (4,3)	348 (4,6)	
Das Kind muss Maske tragen…	Auf dem Schulweg		10.105 (39,0)	668 (16,7)	5704 (39,6)	3733 (49,7)	*p* < 0,0001
	In der Schule außerhalb der Klasse (Gang, Pausenhof)		20.124 (81,1)	1834 (45,8)	12.682 (88,0)	5608 (86,6)	*p* < 0,0001
	In der Schule in der Klasse		12.593 (48,6)	713 (17,8)	6880 (47,8)	5000 (66,5)	*p* < 0,0001
	Im Kindergarten		361 (1,4)	295 (7,4)	53 (0,4)	13 (0,2)	*p* < 0,0001
	In Geschäften		17.763 (68,5)	2060 (51,5)	10.237 (71,1)	5466 (72,7)	*p* < 0,0001
	Nie		1198 (4,6)	933 (24,8)	155 (1,1)	50 (0,7)	*p* < 0,0001
	Hat Attest/Maskenbefreiung		1732 (6,7)	144 (3,6)	1045 (7,3)	543 (7,2)	*p* < 0,0001

*n* = 4427 (17,1 %) fehlende Angabe

*IQR* „interquartile range“

FPP-Masken werden hingegen kaum von Kindern getragen. Auf die Frage, ob Kinder selbst über Beeinträchtigungen durch das Tragen der Maske klagen, antworteten 67,7 % der Eingebenden für ihre Kindern mit Ja; 26 % mit Nein. Die Frage, ob die Eingebenden selber eine Beeinträchtigung des Kindes durch das Tragen der Maske beobachteten, wurde in 66,1 % mit Ja beantwortet (Tab. 2). Die Einschätzung der gesundheitlichen Beeinträchtigung zeigt Tab. 3. Nach der persönlichen Einstellung zu den Coronaschutzmaßnahmen der Regierung gefragt, hatten 4 % keine Meinung. Es gaben 11,7 % der Teilnehmer an, dass die Maßnahmen strenger sein sollten, für 11,0 % fanden die gegenwärtigen Maßnahmen angemessen und gut, und 41,7 % waren für mildere Maßnahmen. Weitere 31,6 % äußerten eine andere Meinung als die in der Auswahl angegebene Einstellungsoption. In der Regel beschrieben diese Teilnehmer die Maßnahmen der Politik als unangemessen, nichtnachvollziehbar und undifferenziert.

**Table 3:** 

	Gesamt, *n* (%)	Altersgruppe 0 bis 6 Jahre, *n* (%)	Altersgruppe 7 bis 12 Jahre, *n* (%)	Altersgruppe 13 bis 17 Jahre, *n* (%)	Chi^2^-Test auf Unterschied
Kopfschmerzen	13.811 (53,3)	960 (24,0)	7863 (54,6)	4988 (66,4)	*p* < 0,0001
Konzentrationsschwierigkeiten	12.824 (49,5)	961 (24,0)	7313 (50,8)	4550 (60,5)	*p* < 0,0001
Unwohlsein	10.907 (42,1)	1040 (26,0)	6369 (44,2)	3498 (46,5)	*p* < 0,0001
Beeinträchtigung beim Lernen	9845 (38,0)	621 (15,5)	5604 (38,9)	3620 (48,2)	*p* < 0,0001
Benommenheit/Müdigkeit	9460 (36,5)	729 (18,2)	5163 (35,8)	3568 (47,5)	*p* < 0,0001
Engegefühl unter der Maske	9232 (35,6)	968 (24,2)	5427 (37,7)	2837 (37,7)	*p* < 0,0001
Gefühl der Atemnot	7700 (29,7)	677 (16,9)	4440 (30,8)	2583 (34,4)	*p* < 0,0001
Schwindel	6848 (26,4)	427 (10,7)	3814 (26,5)	2607 (34,7)	*p* < 0,0001
Trockener Hals	5883 (22,7)	516 (12,9)	3313 (23,0)	2054 (27,3)	*p* < 0,0001
Kraftlosigkeit	5365 (20,7)	410 (10,2)	2881 (20,0)	2074 (27,6)	*p* < 0,0001
Bewegungsunlust, Spielunlust	4629 (17,9)	456 (11,4)	2824 (19,6)	1349 (17,9)	*p* < 0,0001
Jucken in der Nase	4431 (17,1)	513 (12,8)	2550 (17,7)	1368 (18,2)	*p* < 0,0001
Übelkeit	4292 (16,6)	310 (7,7)	2544 (17,7)	1438 (19,1)	*p* < 0,0001
Schwächegefühl	3820 (14,7)	300 (7,5)	2020 (14,0)	1500 (20,0)	*p* < 0,0001
Bauchschmerzen	3492 (13,5)	397 (9,9)	2292 (15,9)	803 (10,7)	*p* < 0,0001
Beschleunigte Atmung	3170 (12,2)	417 (10,4)	1796 (12,5)	957 (12,7)	*p* < 0,0001
Krankheitsgefühl	2503 (9,7)	205 (5,1)	1328 (9,2)	970 (12,9)	*p* < 0,0001
Engegefühl im Brustkorb	2074 (8,0)	161 (4,0)	1122 (7,8)	791 (10,5)	*p* < 0,0001
Augenflimmern	2027 (7,8)	149 (3,7)	1047 (7,3)	831 (11,1)	*p* < 0,0001
Appetitlosigkeit	1812 (7,0)	182 (4,5)	1099 (7,6)	531 (7,1)	*p* < 0,0001
Herzrasen, Herzstolpern Herzstiche	1459 (5,6)	118 (2,9)	766 (5,3)	575 (7,6)	*p* < 0,0001
Rauschen in den Ohren	1179 (4,5)	107 (2,7)	632 (4,4)	440 (5,9)	*p* < 0,0001
Kurzzeitige Bewusstseinsbeeinträchtigung/Ohnmachtsanfälle	565 (2,2)	39 (1,0)	274 (1,9)	252 (3,4)	*p* < 0,0001
Erbrechen	480 (1,9)	40 (1,0)	296 (2,1)	144 (1,9)	*p* < 0,0001

Die Häufigkeitsverteilung der mit Masken assoziierten genannten Nebenwirkungen ist bei den verschiedenen Altersgruppen ähnlich, allen voran Kopfschmerzen, Konzentrationsschwierigkeiten, Unwohlsein, Beeinträchtigung beim Lernen und Benommenheit/Müdigkeit (Tab. 3). Weitere Beschwerden wurden im Freitext beschrieben. Allen voran: 269 Einträge zu verschlechterter Haut, v. a. vermehrte Pickel, Ausschläge und allergische Erscheinungen um den Mundbereich bis hin zu Pilzerkrankungen in und um den Mund. Es gab 151 Einträge zu Nasenbluten, 122 Einträge zu Schulunlust bis hin zu Schulangst/Schulverweigerung, 64 Einträge zu vermehrtem Schwitzen, 52 Eingaben zu Druckstellen und Wunden hinter den Ohren, 46 Eingaben zu wunden oder rissigen und z. T. blutigen Lippen, 31 Einträge zu gesteigerten Migräneanfällen in Frequenz und Ausprägungsgrad, 23 Einträge zu Beeinträchtigungen des Sehens, 13 Einträge zu Aphthen. Die Einstufung einer etwaigen gesundheitlichen Beeinträchtigung der Kinder, wie sie von den Eltern eingeschätzt wurde, zeigt Tab. 3. Weitere Verhaltensauffälligkeiten bei den Kindern, zeigt Tab. 4, allen voran mit 60,4 % eine erhöhte Gereiztheit, 49,3 % weniger fröhliche Kinder, 44 % Kinder, die nicht mehr zur Schule gehen möchten, jeweils sind v. a. Kinder der Alterskategorie 7 bis 12 Jahre betroffen. Bei 25,3 % der Kinder wurde angegeben, dass sie neue Ängste entwickelt haben (Tab. 4). Zudem erwähnen allein 2672 Einträge bei dieser Frage in der Freitexteingabe explizite Spezifizierungen der Angst oder das Neuauftreten mehrerer Ängste. Neben einer allgemeinen Zukunftsangst sind die Ängste, selbst mit Maske zu ersticken sowie vor dem Tod von Angehörigen durch Corona, am häufigsten vertreten. Hinzu kommt die Angst vor Stigmatisierung sowohl durch das Tragen als auch durch das Nichttragen einer Maske im sozialen Umfeld. Viele Eltern berichten auch von Albträumen und Angststörungen, welche sich auf maskierte Menschen beziehen, deren Mimik und Identität für die Kinder nicht erkennbar ist. Eine detaillierte Auswertung und Publikation der Freitexteingaben ist geplant.

**Table 4:** 

	Gesamt, *n* (%)	Altersgruppe 0 bis 6 Jahre, *n* (%)	Altersgruppe 7 bis 12 Jahre, *n* (%)	Altersgruppe 13 bis 17 Jahre, *n* (%)	Chi^2^-Test auf Unterschied
Das Kind ist häufiger gereizt als sonst	11.364 (60,4)	1041 (40,0)	6566 (62,1)	3757 (66,5)	*p* < 0,0001
Das Kind ist weniger fröhlich	9286 (49,3)	959 (36,9)	5640 (53,3)	2687 (47,6)	*p* < 0,0001
Das Kind möchte nicht mehr zur Schule/in den Kindergarten gehen	8280 (44,0)	824 (31,7)	5168 (48,9)	2288 (40,5)	*p* < 0,0001
Das Kind ist unruhiger als sonst	5494 (29,2)	773 (29,7)	3515 (33,2)	1206 (21,4)	*p* < 0,0001
Das Kind schläft schlechter als sonst	5849 (31,1)	633 (24,3)	3507 (33,2)	1709 (30,3)	*p* < 0,0001
Keine weiteren Auffälligkeiten	7103 (27,4)	1400 (35,0)	3834 (26,6)	1869 (24,9)	*p* < 0,0001
Das Kind hat neue Ängste entwickelt	4762 (25,3)	713 (27,4)	2935 (27,8)	1114 (19,7)	*p* < 0,0001
Das Kind schläft mehr als sonst	4710 (25,0)	319 (12,3)	2183 (20,6)	2208 (39,1)	*p* < 0,0001
Das Kind spielt weniger	2912 (15,5)	400 (15,4)	1998 (18,9)	514 (9,1)	*p* < 0,0001
Das Kind hat einen größeren Bewegungsdrang als sonst	1615 (8,6)	253 (9,7)	1124 (10,6)	238 (4,2)	*p* < 0,0001

Die optionale Möglichkeit, Namen und E‑Mail-Adresse für evtl. Rückfragen zu hinterlassen, wurde von 27,1 % (5513) der Teilnehmenden genutzt. Bei der durchgeführten Validierung mit dem Programm „Bouncer“ erwiesen sich 4710 (85,4 %) der E‑Mail-Adressen als erreichbar. Für alle Symptome korrelierte das Vorhandensein von Symptomen mit der Einstellung der Eltern zu den Maßnahmen (*p* < 0,001). Im Folgenden sei ein Beispiel genannt: von den Eltern, die über Kopfschmerzen bei ihren Kindern berichteten, fanden 97, dass die Maßnahmen strenger sein sollten, 7403, dass die Maßnahme milder sein sollten, und 245, dass die Maßnahmen angemessen und gut seien. In Einzelfällen wurden die Teilnehmer auch per E‑Mail kontaktiert, um einzelne Einträge zu validieren.

## Diskussion

Die Brisanz des Themas und das Mitteilungsbedürfnis der Befragten werden durch die „virale“ Nutzung des Registers innerhalb weniger Tage nach Veröffentlichung und durch die, 25.930 Kinder in Deutschland (ca. 2 ‰ der Bevölkerung) betreffenden, Einträge durch Eltern deutlich. Die Tatsache, dass 23,1 % der teilnehmenden Eltern die optionale Möglichkeit, Namen und valide E‑Mail-Adressen für evtl. Rückfragen zu hinterlassen nutzten, ebenso wie die gleichmäßige Verteilung in den Bundesländern, zeugen von der Ernsthaftigkeit der Einträge. Gemäß dem Lagebericht des Robert Koch-Institutes (RKI) vom 25.10.2020 gab es in Deutschland insgesamt 429.181 gemeldete Infektionen, mit steigender Tendenz, von denen 8764 (3,6 % der Gemeldeten) unter 10 Jahre alt und 16.548 (6,7 % der Gemeldeten) zwischen 10 und 19 Jahre alt waren [1]. Das sind weniger als die innerhalb einer Woche in diesem Register gemeldeten Kinder. Ob Kinder eine grundsätzlich geringere Neigung als Erwachsene haben, sich mit SARS-CoV‑2 zu infizieren und diese Infektion auf Erwachsene so zu übertragen, dass Letztere davon schwer krank werden, ist nach wie vor unklar [2–5]. Es zeigt sich jedoch, dass infizierte Kinder, v. a. bis zum 10. Lebensjahr, mehrheitlich keine oder nur milde Symptome entwickeln [6–9]. Selten kommt es bei Kindern bis zum 10. Lebensjahr zu schweren Verläufen. Die bisher, Stand 25.10.2020, einzigen 3 an COVID-19 verstorbenen (bis heute vom RKI nicht genau beschriebenen) Kinder bzw. Jugendlichen hatten chronische Vorerkrankungen [10, 11]. Kinder unter 10 Jahren scheinen in Europa selten Treiber in diesem Infektionsgeschehen zu sein, wobei Daten aus Indien, ein Land mit anderem Hygiene-Hintergrund, durchaus eine gewisse Übertragungsrolle auch Kindern zuschreiben (allerdings ohne Differenzierung zwischen 5‑Jährigen und 17-Jährigen) [12]. Eine schottische Studie an 300.000 Haushalten stellte fest: Je kinderreicher der Haushalt, desto geringer die Wahrscheinlichkeit für eine Hospitalisierung mit COVID-19 bei den Erwachsenen [13]. Eine kürzlich veröffentlichte Studie deutet darauf hin, dass Kinder weniger Aerosol beim Singen und Sprechen emittieren als Erwachsene [14].

Dass das Tragen von Masken bei Erwachsenen grundsätzlich eine sichere, wirksame und kosteneffektive Maßnahme sein kann, um die COVID-19-Pandemie zu verlangsamen, ist unstrittig [15–17]. Anhand der vorliegenden Erhebungen kann jedoch davon ausgegangen werden, dass die Maskenpflicht Auswirkungen auf die Lebensqualität und mutmaßlich auch die Gesundheit einzelner Kinder hat und dies von der Politik und Gesellschaft nicht vernachlässigt werden darf. Während viele Kinder die Maske relativ problemlos vertragen, gibt es klar Kinder, denen eine Maske nicht guten Gewissens zugemutet werden kann, gerade auch bei zusätzlich fraglicher Notwendigkeit des Mund-Nasen-Schutzes bei kleineren Kindern. Ein unsachgemäßer Umgang mit Masken, von dem man bei Kindern tendenziell ausgehen kann, erhöht möglicherweise das Risiko einer Erregerverbreitung und -übertragung durch die gesteigerte Tendenz, sich selbst ins Gesicht zu fassen [18]. Eltern, Lehrer*innen und Ärzt*innen berichten von Stigmatisierung, Ausgrenzung und aggressivem Verhalten gegenüber Kindern, die aus psychischen oder medizinischen Gründen keine Maske tragen. Betrachtet man das Symptomspektrum der Beschwerden, so lässt sich bei 66,1 % der Eingaben eine deutliche und breit gefächerte Beschwerdelast, sowohl im körperlichen (Ausschläge, Kopfschmerzen etc.), wie auch im seelischen (Ängste, Gereiztheit etc.) und im geistigen (Konzentrationsstörung) Bereich bei den Kindern konstatieren. Neben akuten Gesundheitseinschränkungen mit, im Einzelfall als erheblich erlebter, Beeinträchtigung der Gesundheit sind die langfristigen Auswirkungen für die verschiedenen, über das Wohlbefinden hinausgehenden Entwicklungsbereiche, wie beispielsweise Sprache, Spiel, Lernen, Kommunikation, sensomotorische Entwicklung und Empathie von Kindern, schwer abschätzbar. Die häufig genannten Kopfschmerzen und Konzentrationsschwierigkeiten sollten ernsthaft in ihrer Bedeutung für die kognitive Entwicklung erforscht werden. Auffällig ist, dass die Verteilung der Beschwerden gut zu den Lebensaltern passt (Tab. 3 und 4), was die Plausibilität der elterlichen Eingaben unterstützt.

Direkte Auswirkungen der CO_2_-Konzentration in Innenräumen auf die kognitiven Funktionen sind nachgewiesen worden [19, 20]. Dies ist nicht direkt auf die Atemluft unter den Masken übertragbar, doch könnte es unter verschiedenen Maskentypen zu erhöhter CO_2_-Konzentration kommen. Dies gilt womöglich besonders für Stoffmasken, die materialbedingt manchmal eine stärkere Stoffdicke aufweisen und die bei den Kindern im Register mit 65,2 % besonders häufig benutzt wurden. Auch wenn der Maskentyp des Kindes aktuell von den Familien noch im Hinblick auf die Stoffdicke frei gewählt werden kann und somit ein Spielraum zwischen gut atemgängigen und mehrlagigen, eher luftdichten Modellen besteht, bleibt die Problematik bestehen, dass Eltern, ganz unabhängig davon, ob sie selbst die Coronaschutzmaßnahmen befürworten oder nicht, ihre Kinder durch Unwissenheit oder Angst vor Infektion, durch mehrlagige Mundschutze überfordern können. Eine Nutzen-Risiko-Analyse ist also angebracht. Diese ist jedoch dadurch erschwert, dass die Studienlage sowohl hinsichtlich des Nutzens als auch hinsichtlich der Risiken bei Kindern extrem schwach ist. Sowohl die Berechnungen zu einem Nutzen von Masken als auch fast alle Untersuchungen zu den Risiken der Masken beruhen auf Erwachsenen. Es muss auch davon ausgegangen werden, dass die SARS-CoV-2-Schutzstandards für Schulen, wie beispielsweise die der deutschen gesetzlichen Unfallversicherung, nicht überall bekannt sind [21]. Sie beinhalten insbesondere die Empfehlungen zu Erholungszeiten beim Tragen von Masken für Schülerinnen und Schüler mit Kurzpausen und spätestens nach 3 h Tragezeit einer anschließenden Erholungszeit von 15–30 min [21].

Limitierung der Ergebnisse: Auch wenn die rasante Entwicklung des Registers und die hohe Teilnehmerzahl binnen weniger Tage beeindruckend sind, weist diese erste Auswertung des Co-Ki-Masken Registers Limitationen auf. Die Tatsache, dass 38,5 % der Teilnehmenden einen Hochschulabschluss angaben, könnte ein Hinweis sein, dass das Register als Online-Variante und durch die Komplexität nicht allen Personengruppen gleich zugänglich war. Diese Probleme haben alle Online-Register. Ein verzerrtes Berichten auch im Hinblick auf die präferenzielle Dokumentation besonders schwer betroffener Kinder ist nicht auszuschließen. Des Weiteren gelangte der Link zum Register u. a. auch in Social-Media-Foren, welche die Coronaschutzmaßnahmen der Regierung grundsätzlich kritisieren, was sich teilweise in den Ergebnissen zur Abfrage der Einstellung zu den Coronaschutzmaßnahmen der Regierung spiegelt. Zugleich wurde wiederum von anderen Teilnehmern gemeldet, dass ihre Kinder *keine* Beschwerden hatten. Des Weiteren gibt es keine Kontrollgruppe. Die Angaben betreffen Verdachtsfälle von Nebenwirkungen, also medizinische Ereignisse, die im Rahmen der Anwendung von Masken bei Kindern durch die Eltern beobachtet wurden, aber nicht notwendigerweise mit der Maske im Zusammenhang stehen oder von ihr verursacht werden. Sowohl die Geschlechterverteilung als auch die Verteilung der Teilnehmenden nach Bundesländern wie auch die Verteilung der Symptome nach Alter sprechen für eine Repräsentanz der Nutzer. Die Datensätze zeugen in den Freitexteinträgen mit wenigen Ausnahmen von einer sehr differenzierten Betrachtungsweise und ergeben im Ganzen ein ausgewogenes Gesamtbild mit plausiblem Symptomspektrum und einer gut nachvollziehbaren Beschreibung der Beeinträchtigungen, die bei Kindern im Zusammenhang mit der Maske beobachtet werden. Die Beantwortung von Hunderten eingehender E‑Mails durch die Studieninitiatoren mit Fragenbeantwortung zur Existenz des Registers, Spezifizierung und Ergänzung der von Teilnehmenden getätigten Eingaben, ausführlichen Fallschilderungen und Anregungen für weitere Forschung, sind ein weiteres Indiz für die hohe Relevanz des Themas und für die Redlichkeit, mit der viele Teilnehmer sich der Fragestellung widmen. Naturgemäß kann ein offen zugängliches Register niemals alle Eingaben ärztlich gegenvalidieren. Die Registereinträge steigen täglich im mehrstelligen Bereich, und zusätzliche Validitätsprüfungen finden statt, um in absehbarer Zeit weitere belastbarere Daten zur gesundheitlichen Situation von Kindern in Deutschland im Hinblick auf das Tragen eines Mund-Nasen-Schutzes vorlegen zu können. Der Registerfragebogen wird anhand der neuen Symptome, die von den Eltern in den Freitextangaben eingegeben wurden, erweitert und validiert. Beispielsweise sollte das Symptomspektrum um die sogenannte „Maskenrhinitis“ und Nasenbluten erweitert werden.

## Schlussfolgerung

Viele Kinder sind großen Herausforderungen unterworfen, und Familien versuchen, dies bestmöglich zu meistern. Die Zahl der Neuinfektionen ist derzeit auf hohem Niveau. Zumindest für Kinder über 10 Jahre gilt es, die allseits bekannte AHA+L-Regel einzuhalten: Abstand halten, Hygiene beachten, Alltagsmaske tragen sowie regelmäßiges Lüften. In der neuesten Stellungnahme „... zur Verwendung von Masken bei Kindern zur Verhinderung der Infektion mit SARS-CoV-2“ [22] heißt es: „Für Kinder gibt es kaum Daten zu möglichen unerwünschten Wirkungen von Masken“. Das Co-Ki-Masken Register liefert dazu erste Ergebnisse, und diese rufen nach alters- und situationsabhängigen Studien, die eine fundierte Risiko-Nutzen Analyse ermöglichen. Damit zusammenhängend muss auch die Frage nach der Notwendigkeit von Masken-Attesten klinisch und wissenschaftlich bewertet werden.

Sehr wichtig ist uns, dass unsere Ergebnisse nicht dazu führen, dass Eltern grundsätzlich eine negative Meinung zum Maskentragen bei Kindern entwickeln. Viele Kinder und Jugendliche sind dankbar, dass sie dank der AHA+L-Regeln die Schule weiterbesuchen dürfen und würden sich von den Erwachsenen eine positive Meinung zu den Masken wünschen, zumal die Art der getragenen Maske normalerweise gewählt werden kann. Des Weiteren gibt es Kinder, für die die Maske eine notwendige Hilfe sein kann, beispielsweise, wenn sie nach einer Chemotherapie immunsupprimiert sind. Unreflektierte negative Äußerungen über die Maske können ein Nocebo-Effekt herbeiführen und Kinder unnötig belasten: Besser ist es, zuzuhören – und ernst zu nehmen, wenn Probleme auftauchen.

## Fazit für die Praxis

- Dieses weltweit erste Register zu Nebenwirkungen der Maske gibt das Symptomenspektrum bei Kindern und Jugendlichen wieder. Ein gewisser Anteil von Kindern und Jugendlichen scheint nichtzuvernachlässigende Beschwerden beim Tragen der Maske zu haben. Diese Kinder sollten nicht stigmatisiert werden.
- Eine genaue Nutzen-Risiko-Analyse ist dringend angebracht. Das Auftreten von berichteten Nebenwirkungen bei Kindern durch das Tragen der Masken muss ernst genommen werden und braucht eine genaue Abklärung der gesundheitlichen Begleitumstände, der Tragesituation der Maske (Dauer, Pausen und Maskentyp) und der schulischen Situation.
- Weiterhin sind alle Eltern, Ärzt*innen, Pädagog*innen und andere zur Teilnahme an www.co-ki-masken.de eingeladen, ihre Beobachtungen zu Wirkungen, die beim Tragen der Mund-Nasen-Bedeckung auftreten, zu dokumentieren. Das Register steht seit dem 01.12.2020 auch in englischer Sprache zur Verfügung.
- Zurückhaltung mit negativen Äußerungen über die Maske ist angebracht, um Nocebo-Effekte zu vermeiden.

## Abkürzungen

AHA-L: Abstand/Hygiene/Alltagsmaske/Lüften
Co-Ki: Coronakinderstudien
COVID-19: „Coronavirus disease 2019“
SARS-CoV‑2: „Severe acute respiratory syndrome coronavirus 2“(schweres akutes Atemwegssyndrom-Coronavirus Typ 2)
